# Case Management Protocol and Declining Blood Lead Concentrations Among Children

**Published:** 2006-12-15

**Authors:** Nedra S Whitehead, Richard Leiker

**Affiliations:** Research Triangle Institute (RTI) International, Social and Statistical Sciences; Oregon Childhood Lead Poisoning Prevention Program, Environmental and Occupational Epidemiology Section, Oregon Health Services, Office of Disease Prevention and Epidemiology, Portland, Ore

## Abstract

**Introduction:**

Blood lead concentrations among children aged 6 years and younger become a concern at 10 µg/dL (0.48 µmol/L) or higher. The authors' objective was to determine whether initial blood lead concentrations of 10–19 µg/dL (0.48–0.96 µmol/L) declined among children aged 3 years and younger and whether the magnitude of decline was associated with the case management protocol of the state or local childhood lead poisoning prevention program.

**Methods:**

The authors analyzed childhood blood lead surveillance data from 1994 through 1995 and case management protocols from six states that reported the results of all blood lead tests. The study included 2109 children aged 2 years or younger who had a venous blood lead concentration of 10–19 µg/dL (0.48–0.96 µmol/L) and a follow-up venous blood lead test within 3 to 12 months.

**Results:**

Overall, blood lead concentrations increased by 0.25 µg/dL (0.01 µmol/L) between the time of the initial elevated blood lead test and the follow-up test, but concentrations declined by 1.96 µg/dL (0.09 µmol/L) among children covered by a case management protocol that included a home visit and by 0.92 µg/dL (0.04 µmol/L) among those covered by a protocol that included a lead source investigation. The decline remained significant after we adjusted for the child's age.

**Conclusion:**

These findings suggest that childhood lead prevention programs should consider focusing their efforts on home visits and lead source investigations.

## Introduction

Children are exposed to lead from multiple sources, including lead-based paint and lead-contaminated dust. This exposure can have chronic consequences. Preschool children with blood lead concentrations greater than 9 µg/dL (0.43 µmol/L) have lower intelligence and more performance problems on average than do children who are unexposed to lead (1,2). Research by Bellinger et al (1991) and Ruff et al (1996) suggests that scores for children aged 2 years on measures of cognitive performance drop one point for every 1-µg/dL (0.05-µmol/L) increase in blood lead concentration (1,2). Although the decreases in cognitive performance are small individually, in the aggregate they may result in more children with behavioral problems and lower intelligence. Childhood lead exposure can also result in adult chronic health problems (e.g., adverse pregnancy outcomes [3,4], hypertension [5,6]).

In 1991, the Centers for Disease Control and Prevention (CDC) designated blood lead concentrations of 10 µg/dL (0.48 µmol/L) or higher as the level of concern for children aged 6 years and younger. Children were not considered to need environmental or medical intervention unless their blood lead concentration was 20 µg/dL (0.97 µmol/L) or higher (7). An estimated 890,000 children aged 6 years and younger had blood lead concentrations of 10 µg/dL (0.48 µmol/L) or greater in the United States from 1991 through 1994 (8). Although blood lead concentrations among young children have declined since 1994, exposure to lead continues to be a significant problem (9).

Blood lead concentrations of exposed, untreated children increase until children are about aged 2 years and decline thereafter (10). Behavioral and environmental changes to reduce lead exposure are necessary to lower blood lead concentrations before children are aged 2 years. Lead accumulates in bone during chronic exposure and may be released when bone is reabsorbed during pregnancy or lactation (11,12). This release increases maternal blood lead concentrations associated with fetal neurological damage (13,14).

Childhood lead poisoning prevention programs have various case management protocols for children with blood lead concentrations of 10–19 µg/dL (0.48–0.96 µmol/L), but the effect of these case management protocols on children's blood lead concentrations is unknown. We analyzed childhood blood lead surveillance data from six states to examine changes in blood lead concentrations among children aged 2 years and younger to determine whether there was any relationship with case management protocol. We examined the following questions:

- Does blood lead concentration decline after an initial venous blood lead test result of 10–19 µg/dL (0.48–0.96 µmol/L)?
- If so, is the size of the decline associated with state or local case management protocol?
- Does the effect of the case management protocol differ if the initial blood lead concentration is between 10–14 µg/dL (0.48–0.71 µmol/L) or between 15–19 µg/dL (0.72–0.96 µmol/L)?
- Does the effect of case management protocol remain after controlling for a child's demographic characteristics?

## Methods

We defined a case of borderline elevated blood lead concentration as a venous blood lead concentration of 10–19 µg/dL (0.48–0.96 µmol/L), regardless of the case definition used by state and local lead poisoning prevention programs. The case management protocol for a state or county was defined as the method of contact required under the protocol (i.e., mail, telephone, or home visit) and the type of service to be delivered under the protocol (i.e., educational materials on lead exposure prevention alone or lead source investigation) for children with a given blood lead concentration. Each child with blood lead concentrations of 10–19 µg/dL (0.48–0.96 µmol/L) was assigned a method of contact and a type of service according to information on case management protocol provided by the coordinator of each state lead poisoning prevention program. One state, Wisconsin, provided county-level information. Children were assumed to have received the services called for under the case management protocol of their state or county of residence.

Children's demographic information and blood lead test data came from CDC's childhood blood lead database, which is compiled from state childhood blood lead surveillance data (15). Test results are submitted by laboratories and physicians to state surveillance programs, which link the laboratory tests of each child to ensure that duplicate results are deleted and sequential tests are accurately identified. We limited our analysis to six states that 1) had laws requiring that the results of all blood lead tests on children be reported to the state health department, 2) submitted blood lead surveillance data from 1994 and 1995 to CDC, and 3) agreed to have their data used for our analysis. These states were Iowa, Montana, New Mexico, Ohio (only tests performed after April 1995, when universal reporting began, were included), Rhode Island, and Wisconsin.

In 1996, guidelines for blood lead screening changed. Under the new guidelines, targeted screening of children in high risk areas and populations was recommended instead of universal screening — a change that altered the population of children who were tested. We limited our analysis to children who had an initial venous blood lead test concentration of 10–19 µg/dL (0.48–0.96 µmol/L) before they were aged 2 years and who had at least one follow-up venous blood test 3 to 12 months after their initial test. These parameters were selected to ensure sufficient time for interventions to affect blood lead concentrations, to examine the long-term effects of the interventions, and to allow for varied case management protocols. Although a follow-up test within this period was recommended for all children with a venous blood lead concentration of 10–19 µg/dL (0.48–0.96 µmol/L), the timing of the follow-up test varied by case management protocol. The test could be either a venous or a capillary test (7). We limited our analysis to venous tests because greater variability in capillary blood test results made it difficult to identify small changes in blood lead levels. Among the six states, 121,862 children had a blood lead test result in the database, and 4606 (3.8%) children had an initial elevated level between 10–19 µg/dL (0.48–0.96 µmol/L) and at least one follow-up test. Of these children, 2109 (46%) met our study criteria. Our data set included all available test results as well as demographic and intervention data for children who met our study criteria.

We computed the change in blood lead concentration for a child as the difference between the blood lead concentration at the first elevated venous test and the concentration at the first venous follow-up test completed 3 to 12 months after the initial elevated test. We calculated mean changes as the average of individual changes for the group and used paired *t* tests to test for significant changes. We stratified our analysis by blood lead concentration because we expected the magnitude of any decline in blood lead concentration to be related to the initial concentration.

We used Kruskal-Wallis tests to compare mean changes in blood lead concentrations, analysis of variance (ANOVA) to compare the mean number of months needed for blood lead concentrations to decline to less than 10 µg/dL (0.48 µmol/L), and the Mantel-Haenszel chi-square test to determine differences in the proportion of children whose blood lead concentration was less than 10 µg/dL (0.48 µmol/L) at the end of follow-up. We examined the relationship between case management protocol and changes in blood lead concentration over the entire follow-up period and controlled for the age of the child with generalized linear modeling. Quadratic splines with knots at 20, 50, and 80 percentiles for each variable allowed the relationship between a child's age at the initial test, or during the time between tests, and blood lead concentration to differ for different ages or time spans. All the venous blood test results available for a child were used in generalized equalizing equation modeling with autoregressive correlation to examine the longitudinal relationship between case management protocol and blood lead concentration. We controlled for age at the initial and follow-up tests. We used SAS Version 8 (SAS Institute, Inc, Cary, NC) for all analyses (16).

## Results

The children in this study were served by childhood lead poisoning prevention programs that provided parental education by mail (78%), telephone (21%), or home visits (<1%) when a child's blood lead concentration was 10–14 µg/dL (0.48–0.71 µmol/L). Forty-eight percent of children in the study were served by childhood lead poisoning prevention programs that also provided parental education by mail when a child's blood lead concentration was 15–19 µg/dL (0.72–0.96 µmol/L). The other 52% were covered by childhood lead poisoning prevention programs that provided home visits. Eighty-four percent of the children were covered by programs that provided education alone and 16% by programs that provided investigations of the source of lead exposure (Table 1).

Forty percent of children in the study were aged 13 months and younger when they had their first elevated blood lead test result (Table 1). A public agency paid for the initial blood lead test of 33% of the children; 11% had private payers, and payment source was unknown for 56%. Race and ethnicity were known for 68% of the children. Of all children in the study, 29% of the children were white, 21% were black, and 18% were Hispanic. Approximately one half of the children were from Wisconsin.

On average, blood lead concentrations increased by 0.25 µg/dL (0.01 µmol/L) between the first elevated venous blood lead test and the first follow-up test done 3 to 12 months after the initial elevated test. The direction and magnitude of change in blood lead concentration varied by the child's age at the initial elevated test, whether a public or private entity paid for the test, the child's race or ethnicity, and state of residence. Blood lead levels declined, on average, among older children, those whose test was paid for by private funds, those who were not white or Hispanic, and residents of states other than Ohio and Wisconsin. The sex of the child was not associated with changes in blood lead concentrations (Table 1).

Overall, blood lead concentrations declined most among children whose case management protocol called for a home visit (Table 2). Blood lead concentrations decreased by 1.96 µg/dL (0.09 µmol/L) in these children and by 0.72 µg/dL (0.03 µmol/L) among children in families receiving a telephone call as follow-up. In contrast, blood lead concentrations increased 1.18 µg/dL (0.06 µmol/L) among children receiving mail follow-up. Table 3 shows that blood lead concentrations declined by 0.92 µg/dL (0.04 µmol/L), or 6%, on average among children covered by a protocol that included a lead source investigation but rose by 0.36 µg/dL (0.02 µmol/L), or 4%, among children covered by protocols that provided only mailed educational materials.

The contrast between protocols was even more striking for children whose initial blood lead concentration was 10–14 µg/dL (0.48–0.71 µmol/L). Table 2 shows that lead concentrations rose by 1.49 µg/dL (0.07 µmol/L), 13.5%, among children whose parents received mailed materials but declined by 2.20 µg/dL (0.11 µmol/L), 18.8%, among those who received a home visit. Also among children with initial blood lead concentration of 10–14 µg/dL, lead source investigation protocols were associated with an average decline of 1.76 µg/dL (0.08 µmol/L) in lead concentration, whereas education-only protocols were associated with an average increase of 1.07 µg/dL (0.05 µmol/L) (Table 3).

The association with type of contact was less marked for children whose initial blood lead concentration was 15–19 µg/dL (0.72–0.96 µmol/L), and there was no overall difference between blood lead concentrations among children receiving mailed educational materials and those receiving a lead source investigation (Table 3). Blood lead concentrations increased by 0.48 µg/dL (0.02 µmol/L) among children who received mailed materials but declined by 1.95 µg/dL (0.09 µmol/L) among those who received home visits (Table 2). Blood lead concentrations declined by 0.79 µg/dL (0.04 µmol/L) among children who received mailed educational materials only and by 0.72 µg/dL (0.03 µmol/L) among those who received a lead source investigation (Table 3). The association between type of case management protocol and changes in blood lead concentration persisted even after we controlled for a child's age at the first elevated test result and follow-up tests (Tables 2,3). Blood lead concentrations remained lower at the time of later tests among children who received telephone contact or home visits than among those contacted by mail (Table 2). Concentrations also remained lower among those who received a lead source investigation than among those who received mailed educational materials only (Table 3). The results were similar when the analysis was limited to children aged 15 months or younger at the time of their initial blood lead test (data not shown).

Among children with initial blood lead concentrations of 15–19 µg/dL (0.72–0.96 µmol/L) who received a home visit, blood lead concentrations of those who received mailed educational materials declined by 2.49 µg/dL (0.12 µmol/L), a larger decline than that of those who received lead source investigations 0.72 µg/dL (0.03 µmol/L) (data not shown). The difference remained after we adjusted for a child's age but was not significant at later tests.

We did not control for race, ethnicity, and payment source in these models because of missing data; only 23% of records included information on these variables. When we fit models including race and payment source, we found that home visits and lead source investigations among children with initial blood lead concentrations of 10–14 µg/dL (0.48–0.71 µmol/L) were still associated with a significant decline in blood lead concentrations between the time of initial test and first follow-up test, but telephone contact was not significant (data not shown). Including these variables did not change any longitudinal effects significantly.

Blood lead concentrations declined to less than 10 µg/dL (0.48 µmol/L) by the last reported test among 43% of children whose initial blood lead concentration was 10–14 µg/dL (0.48–0.71 µmol/L) and among 23% of children whose initial blood lead concentration was 15–19 µg/dL (0.72–0.96 µmol/L) (Table 4). On average, the time required for blood lead concentrations to drop to less than 10 µg/dL (0.48 µmol/L) was 12 months for children with a concentration of 10–14 µg/dL (0.48–0.71 µmol/L) and 13 months for those with an initial concentration of 15–19 µg/dL (0.72–0.96 µmol/L). Home visits were associated with the highest proportion of children with blood lead concentrations of less than 10 µg/dL (0.48 µmol/L) by the last reported follow-up test and with the shortest average time required for blood lead concentrations to decline to less than 10 µg/dL (0.48 µmol/L).

## Discussion

We found that home visit protocols were associated with a larger decline in blood lead concentrations than mail or telephone contact protocols, regardless of a child's initial blood lead concentration. Mailed educational materials alone were not associated with lower blood lead concentrations.

There are several possible limitations to our study. One is that we may have underestimated the effects of case management protocol. Another is that tested children may not have actually received the services called for by the lead poisoning prevention program in their area. We also had no information on the details of interventions and how these may differ among programs. State laws require the reporting of blood lead tests, but some results, especially those below 10 µg/dL (0.48 µmol/L) or conducted by out-of-state laboratories, may not have been reported or linked to an appropriate child. We used the first venous test performed at least 3 months after the index test of 10–19 µg/dL, but we included children who had at least one follow-up test up to 12 months. Blood lead concentrations among children who had more time between the initial test and the follow-up test would have more time to decline, and this effect could bias our results. The analyses that controlled for the timing of tests, however, should not be affected by this limitation.

The effect of other limitations is less clear. We had no information on many variables, such as iron or calcium intake, that affect blood lead concentrations. Demographic characteristics were missing for many children. We cannot determine how results may have differed if we had been able to control for race and payment source, but results of analyses that included these variables were similar to ones that did not. For these factors to affect our results, however, they would have to be associated with both the case management protocol of the state or county childhood lead poisoning prevention program and with changes in a child's blood lead concentration. Although many factors are associated with an initial elevated blood lead concentration, few have been found to affect changes in blood lead concentration (16-20). Our study was limited to states that require all blood lead test results be reported to the state health department and may not apply to states not included in the analysis.

Surveillance data result from tests performed at the request of a child's parents or physician, and circumstances for these children may differ from circumstances of children who are not tested. This limitation may affect the applicability of our findings to other children. Parents of children who had an elevated blood lead test result but who did not take their children for follow-up tests may be less likely to implement measures to control lead exposure than parents of children who did receive follow-up tests. If this is true, we would expect all case management protocols to be less effective in children overall than we found among children in our study.

The total amount of lead stored in tissues in a child's body can affect blood lead concentrations and obscure the efficacy of interventions. Lead is released during bone turnover until all the stored lead has been released from tissues. Rust et al estimated that among children aged 2 years and younger, bone lead stores could elevate blood lead concentrations for up to 1 year after all sources of lead exposure were removed (21). Bone lead stores could not be estimated for children in this study and may have caused us to underestimate the efficacy of all case management protocols. Differences in bone lead stores, however, could account for our results only if children who received mail or education interventions had higher bone lead stores on average than those of the same age and initial blood lead concentration who received home visits or lead source investigations.

Our study has several strengths in relation to other studies of interventions to reduce blood lead concentrations. We had a large sample, which allowed us to detect small effects on blood lead concentrations, and a diverse study population in terms of race and ethnicity, population density, and geographic region. Our blood lead tests were linked by child and allowed us to compare changes in an individual's blood lead concentration instead of an average for a group.

We identified five randomized trial studies that examined the effectiveness of interventions to reduce blood lead concentrations among children who had concentrations of less than 20 µg/dL (0.97 µmol/L) (17,18,22,23). These interventions included professional house cleaning (17,22), provision of household cleaning supplies and instructions (23), vacuuming with high efficiency particulate (HEPA) air filters (18), and provision of an individualized care plan that included multiple home visits and parental teaching on lead exposure prevention (24). Only one study intervention was effective: repeated professional house cleaning resulted in a 2.1 µg/dL (0.1 µmol/L) decrease in blood lead concentration (22). In four of the studies, however, the controls received a home visit that included information about prevention of lead poisoning or identification of lead hazards; this information was likely similar to information provided by lead poisoning prevention programs (17,18,22,24). The changes in mean blood lead concentration among these control groups varied after 3 months: −5.9 µg/dL (–0.28 µmol/L) (17); −1.0 µg/dL (−0.05 µmol/L) (18), +0.1 µg/dL (<0.01 µmol/L) (22), and −6.2 µg/dL (−0.3 µmol/L) (24). Rhoads et al included information on accident prevention and provided safety equipment in addition to information on lead hazards and lead poisoning prevention. The presence of safety and accident information may have lessened the impact of the lead poisoning prevention information (22). Hilts et al conducted their study near an active lead smelter; this location increased the likelihood of lead exposure. Aschengrau et al had only 24 children in randomized groups and included older children whose blood lead levels may already have begun to decline. The comparison group in the study by Brown et al received one or two educational home visits. In the remaining study, controls received a brochure on lead poisoning prevention (23). The blood lead concentrations of these children declined slightly [0.60 µg/dL (0.03 µmol/L)], but the average age at recruitment was 20 months, so the decline may be attributable to the natural decline as children age.

All of these other studies measured the change in group mean blood lead concentrations. We measured the change in blood lead concentrations for individual children. The decline in blood lead concentrations among children in our study who received home visits [−1.96 µg/dL (0.09 µmol/L)], however, was within the range of changes in blood lead concentrations among these control groups.

Schultz et al, in a study of children followed by a local lead poisoning prevention program, examined the average changes in blood lead concentrations before and after the program implemented home education visits among children who had initial blood lead concentrations of 20–24 µg/dL (0.97–1.16 µmol/L) (25). These authors found that blood lead concentrations declined by 3.9 µg/dL (0.19 µmol/L) on average among children who received home visits but increased by 1.0 µg/dL (0.05 µmol/L) among children who received no follow-up. They may have found a larger decline in blood lead concentrations than we did because the children in their study had higher initial blood lead concentrations than those in our study.

The decline in blood lead concentrations among children covered by a case management protocol that included a home visit was larger and faster than predicted by Neimuth and Schultz for children not receiving interventions (26). For children with the age and initial blood lead concentration ranges of this study, Niemuth and Schultz predicted changes at 12-month follow-up tests that ranged from a 7% increase for children aged 6 months with an initial blood lead concentration of 10 µg/dL (0.48 µmol/L) to an 8.2% decrease for children aged 24 months with an initial blood lead concentration of 15 µg/dL (0.72 µmol/L) (26). We found a 19% decline among children whose initial blood lead concentration was 10–14 µg/dL (0.48–0.71 µmol/L) and who were covered by a case management protocol that included a home visit and a 12% decline among those whose initial blood lead concentration was 15–19 µg/dL (0.72–0.96 µmol/L). Mailed educational materials were associated with a 10% increase in blood lead concentrations; this increase was the same or higher than predicted for children receiving no treatment.

Roberts et al estimated the time that would be required for blood lead concentrations to decline to less than 10 µg/dL (0.48 µmol/L) among children who received no intervention. In our study, the time required for initial blood lead concentrations of 10–14 µg/dL (0.48–0.71 µmol/L) to decline to less than 10 µg/dL (0.48 µmol/L) among children covered by home visit protocols was similar to that estimated by Roberts et al for children with blood lead concentrations in this range who received no intervention (27). Among children whose initial blood lead concentration was 15–19 µg/dL (0.72–0.96 µmol/L) and who received a home visit, however, blood lead concentrations declined to less than 10 µg/dL (0.48 µmol/L) in 10 months — 4 months faster than was estimated by Roberts et al (27).

Some clinicians have questioned the value of following children with blood lead concentrations of less than 15 µg/dL (0.72 µmol/L) (28). Although a systematic evaluation of the effect of different types of programs is needed, our results suggest that blood lead concentrations of less than 20 µg/dL (0.97 µmol/L) can be significantly reduced with protocols that include home visits and lead source investigations. This reduction is valuable because recent research has suggested that even children with blood lead concentrations of less than 10 µg/dL (0.48 µmol/L) have identifiable cognitive deficits associated with lead exposure because stored lead has long-term effects (16,29). Other research has found that strong state policies requiring lead abatement in housing result in fewer children exposed to lead and fewer children with elevated blood lead concentrations (30,31). Home investigations for children with borderline elevated blood lead concentrations, combined with strong lead abatement policies, could lower current and future lead exposure among children.

## Figures and Tables

**Table 1:** Changes in Blood Lead Concentrations Between Initial Blood Lead Test and Follow-up Test by Demographic Characteristics Among Children Aged 2 Years and Younger in Six U.S. States, 1994–1995

Characteristics	No. Children (%)	Mean Change^a^ (µg/dL) (SE)	*t* Test *P *Value	Kruskal-Wallis *P *Value
Total	2109 (100.0)	0.25 (0.2)	.11	—
**Age at first test, mo.**				
0-7	58 (2.8)	2.40 (1.1)	.04	.005
8-10	246 (11.7)	1.34 (0.5)	.009	
11-13	540 (25.6)	0.53 (0.3)	.12	
14-16	431 (20.4)	0.14 (0.3)	.67	
17-19	395 (18.7)	−0.39 (0.4)	.27	
20-24	439 (20.8)	−0.29 (0.3)	.38	
**Sex**				
Male	1054 (50.0)	0.32 (0.2)	.16	.67
Female	937 (44.4)	0.16 (0.2)	.51	
Unknown	118 (5.6)	0.46 (0.7)	.49	
**Payment source for laboratory test**				
Public	697 (33.0)	0.02 (0.3)	.94	<.001
Private	225 (10.7)	−1.31 (0.5)	.005	
Unknown	1187 (56.3)	0.69 (0.2)	.001	
**Race and ethnicity**				
White	610 (28.9)	0.83 (0.3)	.007	.02
Black	434 (20.6)	−0.10 (0.3)	.74	
Native American	15 (0.7)	−1.53 (1.7)	.38	
Hispanic	385 (18.3)	0.50 (0.4)	.18	
Other and unknown	665 (31.5)	−0.14 (0.3)	.65	
**State**				
Iowa	393 (18.6)	1.41 (0.3)	<.001	<.001
Montana	33 (1.6)	3.03 (0.7)	<.001	
New Mexico	16 (0.8)	3.13 (1.2)	.02	
Ohio	224 (10.6)	0.34 (0.4)	.43	
Rhode Island	423 (20.0)	1.09 (0.4)	.004	
Wisconsin	1020 (48.4)	1.6 (0.2)	<.001	

a Blood lead concentrations are expressed in µg/dL. Conversion to SI µmol/L units is µg/dL × 0.0483 = µmol/L.

**Table 2:** Unadjusted and Adjusted Mean Changes in Blood Lead Concentration by Initial Level and Method of Family Contact Among Children Aged 2 Years and Younger in Six U.S. States,^a^ 1994–1995

**Method of Contact for All Children in Study**	No. Children	Mean Change Unadjusted for Age^b^		Mean Change Adjusted for Age^c^		Mean Decline Adjusted for Age^d^	

		µg/dL^e^ (SE)	*t* Test *P* Value	µg/dL (SE)	Wald *P* Value	µg/dL (SE)	Wald *P* Value
**Children with initial blood lead concentration 10-19 µg/dL**	2109	0.25 (0.2)	.11	—	—	—	—
Mail	1383	1.18 (0.2)	<.001	Ref	—	Ref	—
Telephone	262	−0.72 (0.02)	.04	−1.95 (0.5)	<.001	−0.80 (0.3)	.01
Home visit	464	−1.96 (0.4)	<.001	−2.98 (0.4)	<.001	−2.00 (0.2)	<.001
**Children with initial blood lead concentration 10-14 µg/dL**	1233	0.99 (0.2)	<.001	—	—	—	—
Mail	961	1.49 (0.2)	<.001	Ref	—	Ref	—
Telephone	262	−0.72 (0.3)	.04	−2.10 (0.5)	<.001	−1.04 (0.3)	<.001
Home	10	−2.20 (1.6)	.20	−3.68 (2.2)	.09	−0.86 (0.4)	.04
**Initial blood lead concentration 15-19 µg/dL**	876	−0.78 (0.3)	.003	—	—	—	—
Mail	422	0.48 (0.4)	.20	Ref	—	Ref	—
Telephone	0	—	—	—	—	—	—
Home	454	−1.95 (0.4)	<.001	−2.50 (0.6)	<.001	−1.87 (0.3)	<.001

Ref indicates reference group.

a States are Iowa, Montana, New Mexico, Ohio, Rhode Island, and Wisconsin.

b Mean change in blood lead concentration between initial test and final follow-up test 3–12 months after initial test.

c Mean change in blood lead concentration between initial test and first follow-up test 3–12 months after initial test, adjusted for child's age.

d Mean change in blood lead concentration over entire follow-up period, adjusted for child's age at initial and follow-up testing.

e Blood lead concentrations are expressed in µg/dL. Conversion to SI µmol/L units is µg/dL × 0.0483 = µmol/L.

**Table 3:** Unadjusted and Adjusted Mean Changes in Blood Lead Concentration by Case Management Service Among Children Aged 2 Years and Younger in Six U.S. States,^a^ 1994–1995

**Type of Service for All Children in Survey**	No. Children	Mean Change Unadjusted for Age^b^		Mean Change Adjusted for Age^c^		Mean Decline Adjusted for Age^d^	

		µg/dL^e^ (SE)	*t *Test *P* Value	µg/dL^e^ (SE)	Wald *P* Value	µg/dL^e^ (SE)	Wald *P* Value
**Initial blood lead concentration 10-19 µg/dL**							
Education	1939	0.36 (0.2)	.03	Ref	—	Ref	—
Lead source investigation	170	−0.92 (0.5)	.06	−0.93 (0.6)	.12	−0.89 (0.3)	.001
**Initial blood lead concentration 10-14 µg/dL**							
Education	1200	1.07 (0.2)	<.001	Ref	—	Ref	—
Lead source investigation	33	−1.76 (0.7)	.01	−2.59 (1.2)	.03	−1.81 (0.4)	<.001
**Initial blood lead concentration 15-19 µg/dL**							
Education	739	−0.79 (0.3)	.007	Ref	—	Ref	—
Lead source investigation	137	−0.72 (0.6)	.22	0.24 (0.8)	.75	−0.17 (0.3)	.61

Ref indicates reference group.

a States are Iowa, Montana, New Mexico, Ohio, Rhode Island, and Wisconsin.

b Mean change in blood lead concentration between initial test and final follow-up test 3–12 months after initial test.

c Mean change in blood lead concentration between initial test and end of follow-up test period 3–12 months after initial test, adjusted for child's age.

d Mean change in blood lead concentration between initial test and end of follow-up, adjusted for child's age at initial test.

e Blood lead concentrations are expressed in µg/dL. Conversion to SI µmol/L units is µg/dL × 0.0483 = µmol/L.

**Table 4:** Mean Number of Months Required to Reach Blood Lead Concentrations <10 µg/dL by End of Follow-up Period in Six States,^a^ 1994–1995

Protocol	No. Children Who Reached <10 µg/dL	% Children Who Reached <10 µg/dL (SE)	Mean No. Months to reach <10 µg/dL (SE)	*χ* ^2^ *P* Value	ANOVA *P* Value
**Initial blood lead concentration 10-14 µg/dL (N = 1233)**	530	43.0 (1.4)	11.6 (9.9)	—	—
Method of contact					
Mail	353	36.7 (1.6)	12.3 (0.6)	<.001	.06
Telephone	169	64.5 (3.0)	10.3 (0.6)		
Home visit	8	80 (13.3)	8.9 (1.7)		
Case management service					
Education	505	42.1 (1.4)	11.4 (0.4)	.001	.04
Lead source investigation	25	75.8 (7.6)	15.6 (2.4)		
**Initial blood lead concentration 15-19 µg/dL (N = 876)**	205	23.4 (1.4)	12.7(0.8)	—	—
Method of contact					
Mail	67	15.9 (1.8)	18.2 (1.8)	<.001	<.001
Telephone	0	NA	NA		
Home visit	138	30.4 (2.2)	10.0 (0.6)		
Case management service					
Education	184	24.9 (1.6)	13.4 (0.8)	.02	.004
Lead source investigation	21	15.3 (3.1)	6.1 (0.9)		

ANOVA indicates analysis of variance; NA, not applicable.

a States are Iowa, Montana, New Mexico, Ohio, Rhode Island, and Wisconsin.
